# *Ex vivo* peripheral nerve detection of rats by spontaneous Raman spectroscopy

**DOI:** 10.1038/srep17165

**Published:** 2015-11-25

**Authors:** Takeo Minamikawa, Yoshinori Harada, Tetsuro Takamatsu

**Affiliations:** 1 Department of Pathology and Cell Regulation, Graduate School of Medical Science, Kyoto Prefectural University of Medicine, 465 Kajii-cho Kawaramachi-Hirokoji, Kamigyo-ku, Kyoto, 602-8566, Japan; 2 Department of Medical Photonics, Graduate School of Medical Science, Kyoto Prefectural University of Medicine, 465 Kajii-cho Kawaramachi-Hirokoji, Kamigyo-ku, Kyoto, 602-8566, Japan

## Abstract

Nerve-sparing surgery is increasingly being applied to avoid functional deficits of the limbs and organs following surgery. Peripheral nerves that should be preserved are, however, sometimes misidentified due to similarity of shape and color to non-nerve tissues. To avoid misidentification of peripheral nerves, development of an *in situ* nerve detection method is desired. In this study, we report the label-free detection of *ex vivo* peripheral nerves of Wistar rats by using Raman spectroscopy. We obtained Raman spectra of peripheral nerves (myelinated and unmyelinated nerves) and their adjacent tissues of Wistar rats without any treatment such as fixation and/or staining. For the identification of tissue species and further analysis of spectral features, we proposed a principal component regression-based discriminant analysis with representative Raman spectra of peripheral nerves and their adjacent tissues. Our prediction model selectively detected myelinated nerves and unmyelinated nerves of Wistar rats with respective sensitivities of 95.5% and 88.3% and specificities of 99.4% and 93.5%. Furthermore, important spectral features for the identification of tissue species were revealed by detailed analysis of principal components of representative Raman spectra of tissues. Our proposed approach may provide a unique and powerful tool for peripheral nerve detection for nerve-sparing surgery in the future.

Nerve-sparing surgery, which aims to preserve peripheral nerves near the tissue being removed, is now recognized as a highly effective therapeutic procedure that preserves functions of the limbs and organs following surgery and improves the patients’ quality of life. Nerve-sparing surgery has widely been applied in such fields as urological surgery, gynecological surgery, head and neck surgery, and gastrointestinal surgery^1^,^2^,^3^,^4^,^5^,^6^,^7^,^8^,^9^,^10^,^11^,^12^,^13^. In general, assessment by the surgeon’s own eye and/or white-light imaging is applied to identify peripheral nerves. Peripheral nerves that should be preserved are, however, sometimes misidentified due to similarity of shape and color to non-nerve tissues. Electrical stimulation of peripheral nerves can be employed for motor nerve identification^14^,^15^. Electrical stimulation, however, cannot be applied to sensory nerves and autonomic nerves. Dye-based visualization methods are also proposed^16^,^17^. Although dye labeling enhances the visualization contrast of nerves against background tissues, toxicity and nerve specificity are matters of concern for human application. Optical coherence tomography (OCT) and multi-photon microscopy (MPM) were also proposed for peripheral nerve detection^18^,^19^,^20^,^21^,^22^. OCT and MPM detect peripheral nerves by three-dimensional visualization of peripheral nerves based on light interferometry or inherent autofluorescence of peripheral nerves. These techniques, however, provide only morphological information on peripheral nerves due to low molecular sensitivity of these techniques, and as a result final identification of peripheral nerves should be made by a surgeon.

To realize the direct detection of the peripheral nerves on the basis of molecular constituents of peripheral nerves, we have recently proposed a nerve detection technique employing spontaneous Raman spectroscopy^23^. Spontaneous Raman spectroscopy provides information about molecular vibrations that appear in inelastic scattered light of monochromatic excitation light. Since molecular vibration is strongly related to the molecular species and structures of tissues, spectral analysis of inelastic scattered light allows us to identify tissue species via molecular vibrations^24^,^25^,^26^,^27^,^28^. As Raman spectroscopy does not require any treatment such as staining and/or fixation, *ex vivo* and *in vivo* observation of living tissues can be realized noninvasively^29^,^30^,^31^,^32^,^33^,^34^,^35^. Spontaneous Raman spectroscopy and its alternative Raman spectroscopy, coherent anti-Stokes Raman spectroscopy, have been applied to myelinated nerve detection in both the central and the peripheral nervous systems^36^,^37^,^38^,^39^. Unmyelinated nerves that are also important to maintain functions of organs were not visualized. In our previous report, we have provided a proof-of-principle demonstration of selective detection of peripheral nerves including myelinated and unmyelinated nerves against adjacent tissues by using dried microsection samples after a frozen-thawed procedure^23^. In such dried microsection samples, cross-sections of nerve fibers were directly examined by means of Raman spectroscopy. However, *in situ* macroscopic detection must be approached across the surrounding tissues such as the perineurium. The feasibility of our peripheral nerve detection method is still unclear.

In this study, we investigated the feasibility of peripheral nerve detection with Raman spectroscopy by using fresh *ex vivo* macrosamples from Wistar rats. We examined myelinated nerve, unmyelinated nerve, adipose tissue, collagenous tissue, and skeletal muscle without any treatments such as fixation, staining, or microsectioning. A principal component regression (PCR)-based discriminant analysis was performed for the selective detection and further spectral analysis of peripheral nerves against adjacent tissues, and the detection power of peripheral nerves was examined.

## Results

### Raman spectra of nerves and adjacent tissue in fresh *ex vivo* samples

**Figure 1:**
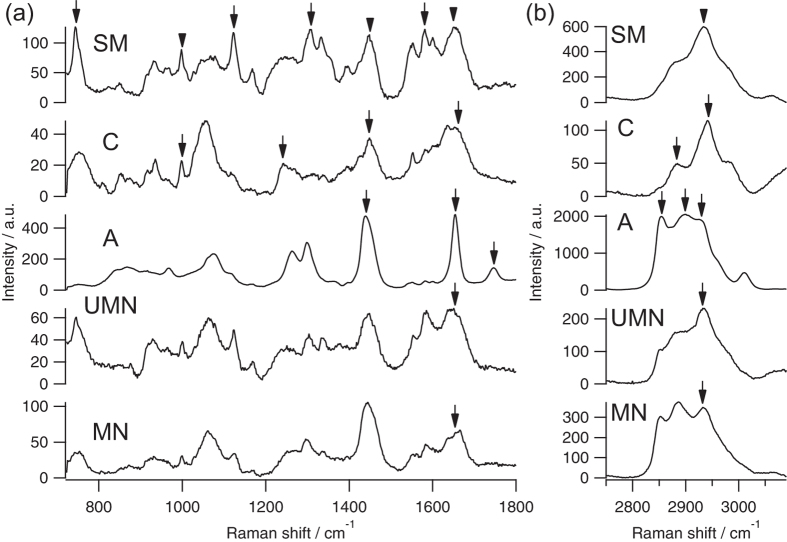
Representative *ex vivo* Raman spectra of myelinated nerve (MN), unmyelinated nerve (UMN), adipose tissue (A), collagenous tissue (C), and skeletal muscle (SM). (**a**) Raman spectra in fingerprint region, and (**b**) in high wavenumber region. a.u., arbitrary units.

For selective peripheral nerve detection, we measured Raman spectra of the peripheral nerves and the predominant contents around peripheral nerves as follows: myelinated nerve, unmyelinated nerve, adipose tissue, collagenous tissue, and skeletal or smooth muscle. We excised representative tissues of these tissue species, *i.e.*, sciatic nerves as myelinated nerve, vagus nerves as unmyelinated nerve, abdominal adipose tissues as adipose tissue, Achilles’ tendon as collagenous tissue, and femoral muscle as skeletal muscle. In our previous study, we found that the Raman spectra of peripheral nerves had almost no site-specific dependency, thus we chose sciatic nerves and vagus nerves as representative peripheral nerves^23^. Our previous study also revealed that the representative Raman spectrum of smooth muscle in blood vessels was very similar to that of skeletal muscle. We thus investigated only the skeletal muscle was investigated in this study. Area-averaged representative Raman spectra of these tissues were obtained *ex vivo*, and are shown in Fig. 1. Since there were no significant Raman bands related to tissues in the Raman spectra between 1800 and 2750 cm^−1^, we showed only the Raman spectra from 725 to 1800 and from 2750 to 3090 cm^−1^ in Fig. 1. We did not find any spectral differences of Raman spectra of respective tissue species among animals. Tissue species of the observed region were confirmed by histological analysis with hematoxylin-eosin staining after Raman observation.

Myelinated nerves and unmyelinated nerves are both important for maintaining organ functions. Myelinated nerves have a myelin sheath that is composed of abundant lipids, whereas unmyelinated nerves have no myelin sheath^40^. This molecular difference mainly appeared at 2850 cm^−1^, assigned to CH_2_ stretching vibration mode. The spectral features of both myelinated and unmyelinated nerves in fresh *ex vivo* macrosamples were similar to those reported in our previous study using dried microsection samples, with Raman bands at 1000, 1126, 1168, 1297, 1336, 1445, 1585, 1654, 2885 and 2932 cm^−1 ^23. In fresh *ex vivo* peripheral nerves, relatively broad Raman bands of amide I appearing at 1654 cm^−1^ as indicated by the arrow in Fig. 1a were obtained. Furthermore, the relative intensity of CH_3_ stretching vibration mode appearing at 2932 cm^−1^ as indicated by the arrow in Fig. 1b was relatively strong comparing with that observed in our previous study using dried macrosection samples. These results were different from those seen in Raman spectra of dried microsection samples, and thus indicated that the protein contributions in fresh *ex vivo* peripheral nerves were relatively large. In our previous study with dried microsection samples, we directly observed peripheral nerves^23^. In this study with fresh *ex vivo* peripheral nerves, we observed the peripheral nerves across the perineurium that mainly comprises collagens, which might result in the Raman features of the protein contributions that appeared in the fresh *ex vivo* samples.

Adipose tissue, collagenous tissue, and skeletal muscle are predominant components around peripheral nerves. In adipose tissue, spectral features appeared at 1080, 1260, 1299, 1441, 1655, 1747, 2855, 2899 and 2930 cm^−1^. The adipose tissue was composed of fat cells with large lipid droplets that occupied most regions of the fat cells. The lipid droplets were predominantly composed of triglycerides, and thus the Raman bands of adipose tissue are mainly assigned to lipid-related bands such as CH_2_ bending mode (1441 cm^−1^), C = C double bond stretching mode (1655 cm^−1^), C = O double bond stretching mode (1747 cm^−1^), CH_2_ stretching modes (2855 and 2899 cm^−1^), and CH_3_ stretching mode (2930 cm^−1^) as indicated by the arrows in Fig. 1a,b. In skeletal muscle, the main Raman bands appeared at 746, 1000, 1124, 1309, 1333, 1450, 1550, 1581, 1650, 2883 and 2934 cm^−1^. Especially relatively strong Raman bands at 746, 1124, 1309 and 1581 cm^−1^ as indicated by the arrows in Fig. 1a might be assigned to reduced cytochromes present in mitochondria in skeletal muscle cells^41^,^42^. The Raman bands that related to cellular components such as phenylalanine (1000 cm^−1^), CH_2_ bending mode (1450 cm^−1^), Amide I (1650 cm^−1^), and CH_3_ stretching modes (2934 cm^−1^) as indicated by the arrowheads in Fig. 1a,b were also appeared. For investigation of collagenous tissue, we obtained Raman spectra of Achilles’ tendon. Collagenous tissue is generally present around peripheral nerves, *e.g.*, perineurium and fibrous connective tissue; complete surgical isolation of perineurium and fibrous connective tissue in macrosamples was difficult due to their small size. Our previous study revealed that fibrous connective tissue surrounding peripheral nerves is mainly composed of collagen type-I^23^. Therefore, we used tendon that is predominantly composed of collagen type-I as a model tissue of collagenous tissue. The main Raman bands of collagenous tissue appeared at 812, 853, 935, 1000, 1030, 1241, 1448, 1662, 2883 and 2943 cm^−1^. Especially, 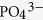 (1000 cm^−1^), Amide III (1241 cm^−1^), CH_2_ bending mode (1448 cm^−1^), Amide I (1662 cm^−1^), CH_3_ symmetric stretching mode (2883 cm^−1^), and CH_3_ symmetric and CH_2_ asymmetric stretching modes (2943 cm^−1^) as indicated by the arrows in Fig. 1a,b were representative Raman bands related to collagen type-I.

### Principal component analysis of Raman spectral data set

**Figure 2:**
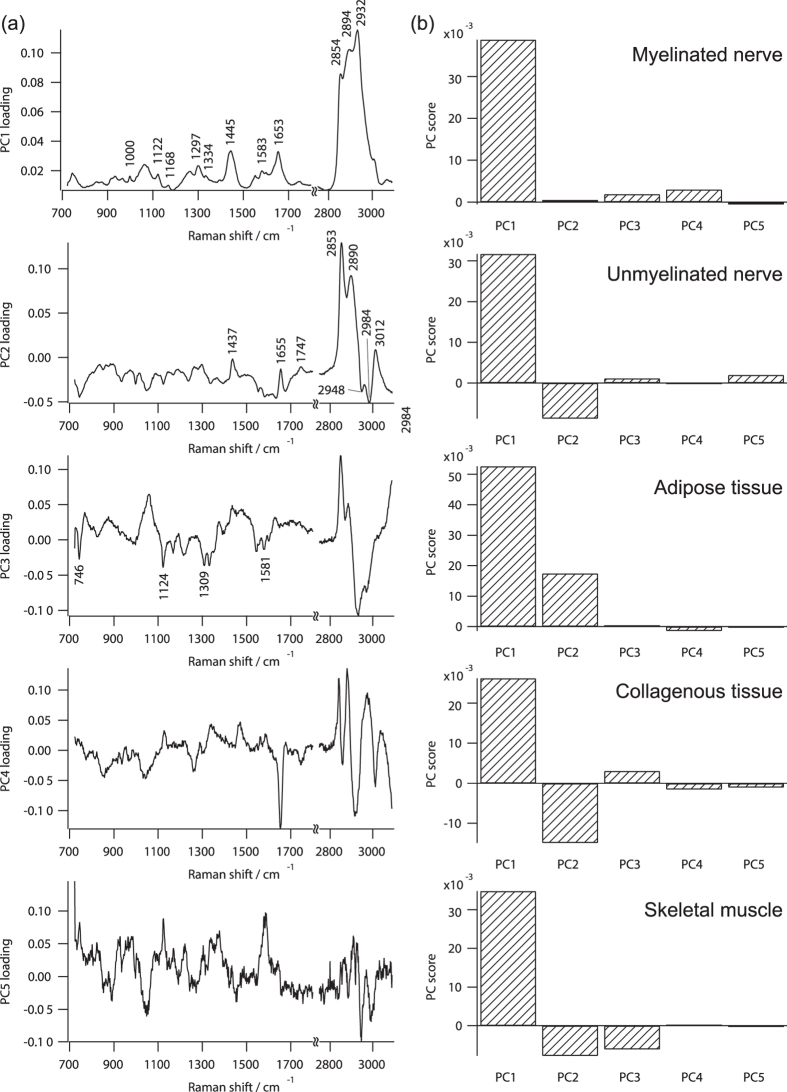
(**a**) Loading and (**b**) score plots of Raman spectra of tissue species Sbase calculated by principal component analysis.

We constructed a prediction model based on PCR-based discriminant analysis. We first performed principal component analysis using the representative Raman spectra of myelinated nerve, unmyelinated nerve, adipose tissue, collagenous tissue, and skeletal muscle as shown in Eq. 2 (see Materials and Methods). We employed both Raman spectra of fingerprint region and high wavenumber region to realize reliable prediction as discussed in our previous study^23^. To simply elucidate the differences of Raman spectra among tissue species, we performed principal component analysis with the representative Raman spectra of tissue species. Loading and score plots of the representative Raman spectral data set are shown in Fig. 2. In our model, we used 5 Raman spectra derived from 5 tissue species in *S*_base_, and thus a total of 5 principal components (PCs) were calculated. Contribution ratios of 1st PC (denoted as PC1; similar notations for other PCs), PC2, PC3, PC4, and PC5 were 90.7%, 8.38%, 0.655%, 0.171%, and 0.0701%, respectively.

In the score plot related to the Raman spectrum of myelinated nerve, PC1 has the largest score, where the absolute score values of the other PCs were less than 10% of PC1. This result indicated that the loading plot of PC1 predominantly had the spectral features of the Raman spectrum of myelinated nerve, *e.g.* 1000, 1122, 1168, 1297, 1334, 1445, 1583, 1653, 2854, 2894 and 2932 cm^−1^. The relative intensity ratio in the CH region (2800–3090 cm^−1^) was slightly different from the representative Raman spectrum of myelinated nerve shown in Fig. 1. To reconstruct the Raman spectrum of myelinated nerve with proper intensity ratio in the CH region, other PCs were needed, especially PC3 and PC4.

In the score plot related to the Raman spectrum of unmyelinated nerve, PC1 had the highest score in the positive direction, and PC2 was the predominant component in the negative direction. This result indicated that the positive peaks appearing in the loading plot of PC2 did not contain or had small contributions in comparison with those of PC1 in the Raman spectrum of unmyelinated nerve. In contrast, the negative peaks appearing in the loading plot of PC2 were emphasized or appeared in the Raman spectrum of unmyelinated nerve. In the comparison between the score plot of the myelinated nerve and that of unmyelinated nerve, PC2 showed the major difference. This result indicated that the loading plots of PC2 might contain features of spectral difference between the Raman spectrum of myelinated nerve and that of unmyelinated nerve. The loading plot of PC2 contained positive peaks at 2853 cm^−1^ and 2890 cm^−1^, indicating the difference of CH_2_ vibration modes between the Raman spectra of myelinated nerve and unmyelinated nerve. The negative peaks at 2948 and 2984 cm^−1^ and the positive peak at 3012 cm^−1^ might reflect in detail the difference of CH_3_ vibration modes between them. Furthermore, there was a peak at 1655 cm^−1^ in the positive direction. This result might indicate that the C = C double bond stretching mode (1655 cm^−1^) of lipids in the myelin sheath was contained in the Raman spectrum of myelinated nerve. This difference was not apparent in the representative Raman spectra due to the broad spectrum of Amide I.

In the score plot related to the Raman spectrum of adipose tissue, PC1 and PC2 were the predominant components. Several peaks assigned to triglycerides of lipid droplets appeared in the positive direction of the loading plot of PC2, including 1655 (C = C double bond stretching mode), 1747 (C = O band carbonyl stretching mode), and 2853 cm^−1^ (CH_2_ symmetric stretching mode)^43^. Furthermore, the peak at 1437 cm^−1^ in the loading plot of PC2 indicated a spectral feature of peak shift of CH_2_ bending mode appearing around 1450 cm^−1^ to lower wavenumber in the Raman spectrum of adipose tissue compared with those of the other tissues. Actually, the score plots of PC2 of myelinated nerve and adipose tissue had positive value, while those in the other tissues had negative value. These results indicated that the spectral features of lipid-containing tissues, such as myelinated nerve and adipose tissue, appeared in the positive peaks of PC2.

In the score plot related to the Raman spectrum of collagenous tissue, PC1 was the predominant component in the positive direction, and PC2 was that in the negative direction, similar to the score plot related to the Raman spectrum of unmyelinated nerve. The score plots related to the Raman spectra of unmyelinated nerve and of the collagenous tissue differed in that the relative values of the score plots of PC2 and PC3 in the collagenous tissue were slightly larger than those in the unmyelinated nerve. Furthermore, the score plots of PC4 and PC5 had negative values in the collagenous tissue, while the score plots of PC4 and PC5 respectively were almost zero and had positive value in the unmyelinated nerve.

In the score plot related to the Raman spectrum of skeletal muscle, PC1 was the predominant component in the positive direction, and PC2 and PC3 were the predominant components in the negative direction. The score plot of PC3 for the skeletal muscle was negative, while the score plots of PC3 in the other tissues were positive or almost zero. This result suggests that the negative peaks on the loading plot of PC3 indicated spectral features of skeletal muscle, such as characteristic Raman bands of cytochromes (746, 1124, 1309, and 1581 cm^−1^).

### Regression coefficient matrix for PCR-based prediction

**Figure 3:**
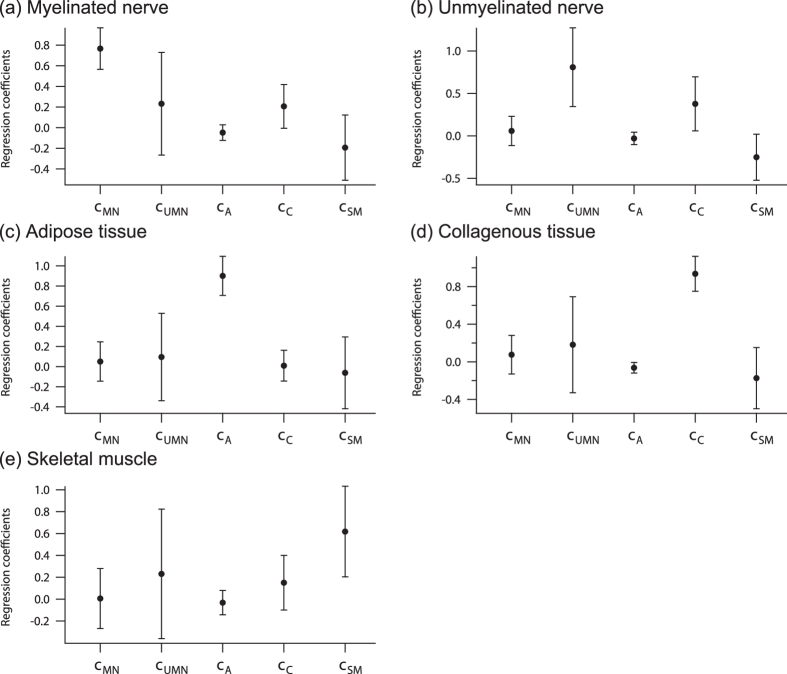
Regression coefficient plots for (**a**) myelinated nerve, (**b**) unmyelinated nerve, (**c**) adipose tissue, (**d**) collagenous tissue, and (**e**) skeletal muscle. Each error bar indicates standard deviation. (**c**) indicates each coefficient. MN, myelinated nerve; UMN, unmyelinated nerve; A, adipose tissue; C, collagenous tissue; and SM, skeletal muscle.

For the prediction of tissue species, we calculated regression coefficient matrix *C* by Eq. 3 (see Materials and Methods). Mean regression coefficients calculated with 1000 observation points (one pixel obtained from a line shaped spectral-image was defined as an observation point) are shown in Fig. 3. The regression coefficient matrices were calculated without the dimensional reduction of PCs. For the myelinated nerve and unmyelinated nerve, the regression coefficient corresponding to the Raman spectrum of each type of nerve exhibited the highest value. The regression coefficient corresponding to the Raman spectrum of collagenous tissue had also relatively large value in the myelinated nerve and the unmyelinated nerve. The value of the regression coefficient indicated the relative contribution of the base spectral data set *S*_base_ in the observed spectrum used in Eq. 3. In our experiments, we obtained Raman spectrum of fresh *ex vivo* peripheral nerve by illuminating from the lateral side, and thus the contribution of the perineurium must be included. The perineurium is predominantly composed of collagenous tissue, and therefore the contribution of collagenous tissue appeared in the regression coefficients for the myelinated nerve and the unmyelinated nerve. The other tissues, such as adipose tissue, collagenous tissue, and skeletal muscle, predominantly had the regression coefficients of their own tissue, respectively. Thus, the regression coefficients corresponding to each tissue type exhibited the highest value, and the other coefficients were much smaller than the regression coefficients of corresponding tissues.

### Prediction capability of the PCR-based prediction model

By using the regression coefficient matrix, we obtained a prediction model by using quadratic discriminant analysis. Cross-validated sensitivities and specificities of the prediction model without dimensional reduction of PCs are shown in Table 1. The PCR-based prediction model realized sensitivities of 95.5%, 88.3%, 96.5%, 89.2%, and 88.2% and specificities of 99.4%, 93.5%, 100%, 98.0%, and 98.6%, respectively, for the prediction of myelinated nerve, unmyelinated nerve, adipose tissue, collagenous tissue, and skeletal muscle.

We also estimated cross-validated sensitivities and specificities with dimensional reduction of each PC (Table 2 and Supplementary Table S1). In the dimensional reduction of PC1, in which PC2, PC3, PC4, and PC5 were employed for the estimation of regression coefficient in Eq. 3 (see Materials and Methods), notable decrease of sensitivity and specificity compared with the sensitivity and specificity obtained without dimensional reduction of PCs was not obtained, indicating that PC1 was not critical for the prediction of tissue species in this model.

In the dimensional reduction of PC2, we obtained notable decrease of the sensitivity for adipose tissue, but not for the other tissues. This result indicated that the peaks appearing in the loading plot of PC2, such as lipid-related peaks at 1437, 1655, 1747, and 2855 cm^−1^, are important for the prediction of adipose tissue, and had insignificant effect on the prediction of the other tissues.

In the dimensional reduction of PC3, notable decrease of sensitivity was obtained for the sensitivities for myelinated nerve, unmyelinated nerve, and skeletal muscle. Especially, the sensitivity for skeletal muscle was decreased to almost half of that in the case without dimensional reduction. This result indicated that the most important components of the Raman spectrum for skeletal muscle for selective prediction appeared in the loading plot of PC3, such as characteristic Raman bands of cytochrome c (746, 1124, 1309, and 1581 cm^−1^). According to the result that the sensitivities for myelinated nerve and unmyelinated nerve were also decreased, the loading plot of PC3 also comprised spectral features for selective prediction of myelinated nerve and unmyelinated nerve.

In the dimensional reduction of PC4, notable decrease was obtained for the sensitivities for myelinated nerve and unmyelinated nerve, while no such decrease was noted for the sensitivity for peripheral nerve, when both myelinated and unmyelinated nerves were considered to be peripheral nerves. In contrast, in the dimensional reduction of PC3, the sensitivity for peripheral nerve was decreased following the decrease of the sensitivities for myelinated nerve and unmyelinated nerve. These results indicated that the loading plots of PC3 and PC4 respectively included spectral features for the prediction of peripheral nerves against the other tissues and for that of each peripheral nerve type.

In the dimensional reduction of PC5, notable decrease was obtained for the sensitivity for unmyelinated nerve. The sensitivity for peripheral nerve was also decreased, indicating that the loading plot of PC5 is comprised of the important spectral features for the prediction for unmyelinated nerve against other non-peripheral nerve tissue species. Actually, most of the mispredictions for unmyelinated nerve were collagenous tissue (data not shown). Although the contribution ratio of PC5 was only 0.0701%, PC5 might be comprised of important spectral features for the prediction of unmyelinated nerve against collagenous tissues.

## Discussion

Our recent study demonstrated the proof-of-principle of Raman spectroscopy for the selective detection of peripheral nerves by using dried microsections after a frozen-thawed procedure^23^. Here, we extended the detection to *ex vivo* spectral analysis of fresh tissue macrosamples, and performed selective detection of peripheral nerves against adjacent tissues and further spectral analysis of fresh *ex vivo* tissues by means of a PCR-based prediction model. Although surgically excised fresh *ex vivo* tissues were used for the experiments in the present study, sample conditions were almost identical to *in vivo* conditions. The result presented in this study suggests that the Raman spectroscopic prediction of peripheral nerves has potential for application in intraoperative use. Raman spectroscopy offers the advantages of fast, *in situ*, non-destructive, and molecular vibration-based observation, enabling the selective detection of peripheral nerves against adjacent tissues without any treatment such as fixation or staining.

It is considered that the most appropriate cases for application of this method might be the detection of small peripheral nerves ranging from 100 μm to 1 mm in diameter. In general, peripheral nerves larger than 1 mm innervate a well-known course with relatively small individual variability, and most of the peripheral nerves can be identified by the human eye or under white-light imaging. In contrast, in the case of peripheral nerves smaller than 100 μm, organ functions generally recover by regeneration of peripheral nerves even if these nerves are disrupted^44^. However, the actual size at which peripheral nerves should be preserved is still unknown. This is because there is no quantitative and intraoperative identification method of small peripheral nerves in current surgery, and surgical damage of peripheral nerves is estimated based on only post-operative recovery of the patient’s nerve function without the evaluation of resection rate of small peripheral nerves. Use of our proposed method offers the potential of revealing actual peripheral nerves that should be preserved by quantitative evaluation of the resection rate of peripheral nerves.

There are some limitations of this study. Firstly, the estimated size of peripheral nerves used in this study was limited. As myelinated nerve, we examined the sciatic nerve of Wistar rats, which ranges from a few tens of micrometers to millimeters in diameter. However, we used vagus nerve for unmyelinated nerve, which is only less than 100 μm in diameter. The other peripheral unmyelinated nerves are narrower than the vagus nerve in the Wistar rat. For further confirmation of this method by using larger peripheral unmyelinated nerves ranging from 100 μm to 1 mm in diameter, larger animals such as the rabbit and pig, and also humans should thus be used. We should also evaluate the effect of the thickness of perineurium depending on the size of the peripheral nerves. Secondly, the toxicity of light irradiation to peripheral nerves is still unclear. In this study, we observed no serious damage of the peripheral nerves and other tissues, such as charring, burning, or structural distortion. For the determination of the adequate irradiation condition including laser power, irradiation area, and exposure time in the observation of Raman spectra of peripheral nerves, further studies are required that include follow-up evaluation of organ functions after surgical use *in vivo*. Thirdly, the optimum excitation wavelength for the peripheral nerve detection with Raman spectroscopy is still unclear. High signal intensity of Raman scattering could be obtained by using short excitation light such as the 532-nm light that we employed in this study due to high Raman scattering coefficient of tissues and the availability of a high quantum efficiency detector, although near-infrared light is better for decreasing autofluorescence, absorption, and Rayleigh scattering of tissues. Furthermore, potential phototoxicity of excitation light to samples is also a matter of concern. For clinical use, we should investigate the effective excitation light for peripheral nerve detection with Raman spectroscopy by using actual peripheral nerves of patients in future studies. Fourthly, the penetration depth of Raman spectroscopy using 532 nm excitation light was limited to several tens to a few hundreds of a micrometer due to the scattering of light in turbid media^45^. The penetration depth might be sufficient because the applicable diameter of peripheral nerve using this method ranges from 100 μm to 1 mm, of which the perineurium might range from a few tens to a few hundreds of a micrometer, as discussed above. If required, we can apply the following methods for further enhancement of penetration depth: use of longer wavelength of excitation such as 780 nm, and application of spatially offset Raman spectroscopy^46^,^47^,^48^. These methods can enhance the penetration depth of Raman spectroscopy to a few times to tens of times.

## Conclusions

In the present study, we demonstrated label-free and selective detection of fresh *ex vivo* peripheral nerves of Wistar rats by using Raman spectroscopy with PCR-based discriminant analysis. By using PCR-based discriminant analysis with representative Raman spectra of peripheral nerves and their adjacent tissues, we revealed important spectral features for the prediction of tissue species. We also successfully and selectively detected peripheral nerves including myelinated nerves and unmyelinated nerves with high sensitivity and specificity. Although for the application of our proposed method to clinical use, further development of an intraoperative Raman spectroscopy system is required, our proposed approach may provide a unique and powerful tool for peripheral nerve detection for nerve-sparing surgery in the future.

## Materials and Methods

### Raman spectroscopy

A slit-scanning confocal Raman microscope (RAMAN-11; Nanophoton, Osaka, Japan) was used for acquiring Raman spectra and Raman spectral images, as described in our previous studies^23^,^30^,^49^,^50^. A frequency doubled Nd:YAG laser (532 nm) was employed for excitation. The excitation laser beam was focused into a line (735 μm in length and approximately diffraction limit in width) on a sample through a cylindrical lens and a long working distance objective lens (UPlanFL N, ×10, NA = 0.3; Olympus, Tokyo, Japan). Raman scattering was collected with the same objective lens, focused onto the input slit of the spectrometer with a 600 grooves/mm grating, and detected with a two-dimensional image sensor (Pixis 400BR, −70 °C, 1340 × 400 pixels; Princeton Instruments, Trenton, NJ, USA). Owing to the line shaped focus of the excitation beam, a one-dimensional spectral image from the line-illuminated site was collected with the same objective lens (one pixel in space corresponds to 1.8 μm in length and approximately 0.89 μm in width). The excitation laser power was 235 μW/μm^2^ on the sample plane. The exposure time for each line and the slit width of the spectrometer were respectively set at 10 s and 70 μm (corresponding spectral resolution was 7 cm^−1^) for all experiments.

### Animals

**Figure 4:**
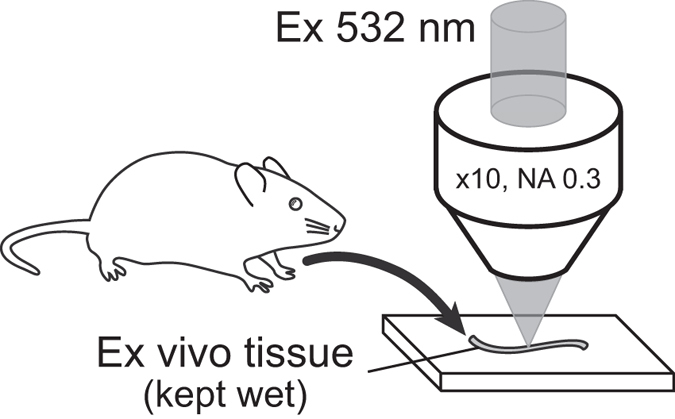
Experimental conditions for *ex vivo* Raman spectroscopy.

All animal experiments were conducted with the approval of and in accordance with guidelines from the Committee for Animal Research, Kyoto Prefectural University of Medicine. Sciatic nerves (1 cm in length), vagus nerves (1 cm in length), abdominal adipose tissues (approximately 1 × 1 × 1 cm^3^), Achilles’ tendon (0.5–1 cm in length), and femoral muscle (approximately 1 × 1 × 1 cm^3^) were excised from male Wistar rats (8–10 weeks old) after euthanasia. A total of 5 rats were used for analysis. One sample of respective tissue species from each rat was studied. The excised fresh tissues were put on a cover slip (No.1; Matsunami Glass Ind., Ltd., Osaka, Japan), and were kept wet throughout the experiment with physiological saline. A schematic diagram of *ex vivo* Raman spectroscopy is shown in Fig. 4.

### Histological examination

For the confirmation of histology of excised tissues, we performed hematoxylin-eosin staining. The excised tissues were embedded in frozen section compound (FSC 22; Leica, Wetzlar, Germany) after Raman observation. The embedded samples were snapfrozen in dry ice-acetone, and then stored at −80 °C. The frozen samples were sliced into 5-μm-thick sections with a cryostat microtome (CM1900; Leica, Wetzlar, Germany). The sections were fixed with 10% formalin, and stained with hematoxylin-eosin.

### Spectral preprocessing

Nanophoton’s software was utilized for preprocessing individual Raman spectra. Wavenumbers of all Raman spectra were calibrated by using the known Raman bands of ethanol. To extract the Raman spectrum from broad fluorescence background, we applied modified polynomial curve fitting method^51^. We estimated the autofluorescence component superposed on the Raman spectrum by calculating a modified least-squares fifth-order polynomial curve with 10 iterations, and then subtracted this polynomial from the raw spectra.

### Construction of prediction model using principal component regression-based discriminant analysis

We employed PCR for the construction of a prediction model of tissues. The PCR is one of the common statistical methods, which provides linearly independent spectral datasets called principle components (PCs). In spectral analysis, linearly independent spectra are useful for extracting spectral features owing to its independency of each spectral component. In this study, PCR was performed by using Matlab (Mathworks, Natick, MA, USA). Firstly, we assumed that the observed Raman spectrum *S*_tissue_ could be expressed by user-defined Raman spectra of tissue species *S*_base_ with regression coefficient matrix *C*:


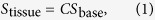


where *S*_tissue_ and *S*_base_ were normalized with the square root summation of their Raman spectra ranging from 725 cm^−1^ to 3090 cm^−1^. In this study, we assumed that S_base_ was the spectral data set of representative Raman spectra of myelinated nerve, unmyelinated nerve, collagenous tissue, adipose tissue, and skeletal muscle, which were normalized with the total intensity of the wavenumber region ranging from 725 cm^−1^ to 3090 cm^−1^. The spectral data set of *S*_base_ was decomposed to loading matrix *P* and score matrix *T* with principal component analysis:


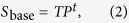


where *P* was orthonormal basis. Then, we calculated the regression coefficient matrix with Moore-Penrose pseudo inverse:


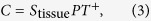


where *T*^+^ indicated the Moore-Penrose pseudo inverse of *T* matrix. Since data points of discrete Raman spectra obtained with the CCD camera in the spectrometer (1300 pix) were much larger than the number of assumed tissue species in *S*_base_ (5 tissue species), Eq. 3 reflects the most reliable regression coefficient matrix with the square root sum of the prediction error. To obatain a reliable *PT*^+^, we used area-averaged representative Raman spectra with high signal-to-noise ratio for *S*_base_ (400–3000 pixels averaging).

By using the regression coefficient matrix *C*, we constructed a prediction model with discriminant analysis. Quadratic discriminant analysis was used for the prediction. A total of 1000 Raman spectra in each tissue species was obtained from 5 rats as training data sets for prediction. Detection sensitivity and specificity were calculated by leave-one-out cross-validation with these data sets.

## Additional Information

**How to cite this article**: Minamikawa, T. *et al.* *Ex vivo* peripheral nerve detection of rats by spontaneous Raman spectroscopy. *Sci. Rep.* **5**, 17165; doi: 10.1038/srep17165 (2015).

## Supplementary Material

Supplementary Information

## Figures and Tables

**Table 1:** Prediction result for each tissue species by using the PCR-based prediction model.

Prediction	Histology					PN (MN+UMN)
	MN	UMN	A	C	SM	
MN	955	15	7	0	2	1884
UMN	31	883	25	105	100	
A	0	0	965	0	0	0
C	14	51	0	892	16	65
SM	0	51	3	3	882	51
Sensitivity (%)	95.5	88.3	96.5	89.2	88.2	94.2
Specificity (%)	99.4	93.5	100	98.0	98.6	92.0
PPV (%)	97.5	77.2	100	91.7	93.9	88.7
NPV (%)	98.9	97.0	99.1	97.3	97.1	96.0

The right column PN (MN+UMN) indicates the sensitivities and specificities in which the peripheral nerves including myelinated nerve and unmyelinated nerve were predicted to be at least either myelinated nerve or unmyelinated nerve. MN, myelinated nerve; UMN, unmyelinated nerve; A, adipose tissue; C, collagenous tissue; SM, skeletal muscle; PN, peripheral nerve; PPV, positive predictive value; and NPV, negative predictive value.

**Table 2:** Prediction results for tissue species with and without dimensional reduction of PCs.

		MN	UMN	A	C	SM	PN (MN+UMN)
w/o DR	Sensitivity (%)	95.5	88.3	96.5	89.2	88.2	94.2
	Specificity (%)	99.4	93.5	100	98.0	98.6	92.0
DR of PC1	Sensitivity (%)	95.7	85.4	96.2	88.1	89.4	92.8
	Specificity (%)	99.4	93.5	100	97.8	98.0	92.0
DR of PC2	Sensitivity (%)	95.6	82.0	82.5	88.1	84.5	90.9
	Specificity (%)	99.2	91.3	98.2	96.2	98.3	88.8
DR of PC3	Sensitivity (%)	88.4	76.2	97.8	88.2	46.7	86.5
	Specificity (%)	96.9	89.1	100	94.7	93.7	84.1
DR of PC4	Sensitivity (%)	84.4	72.9	97.0	90.2	87.3	93.8
	Specificity (%)	95.8	90.7	100	97.9	98.6	92.0
DR of PC5	Sensitivity (%)	95.7	62.2	96.4	82.3	88.5	82.2
	Specificity (%)	98.9	95.0	100	91.4	96.0	94.0

Underlined values are notable difference of prediction sensitivity owing to dimensional reduction. The right column PN (MN+UMN) indicates the sensitivities and specificities in which the peripheral nerves including myelinated nerve and unmyelinated nerve were predicted to be at least either myelinated nerve or unmyelinated nerve. MN, myelinated nerve; UMN, unmyelinated nerve; A, adipose tissue; C, collagenous tissue; SM, skeletal muscle; PN, peripheral nerve; DR, dimensional reduction; and w/o, without.
