# Supplementary material for: Ex vivo peripheral nerve detection of rats by spontaneous Raman spectroscopy
Source: Sci Rep. 2015 Nov 25;5:17165. doi: 10.1038/srep17165 (PMC4658536; doi:10.1038/srep17165)
Supplement: Supplementary Information [file srep17165-s1.pdf]

Ex vivo peripheral nerve detection of rats by spontaneous Raman spectroscopy

Takeo Minamikawa[a,*], Yoshinori Harada[a], and Tetsuro Takamatsu[b]

[a]Department of Pathology and Cell Regulation, Graduate School of Medical Science, Kyoto Prefectural University of Medicine, 465 Kajii-cho Kawaramachi-Hirokoji, Kamigyo-ku, Kyoto, 602-8566, Japan

[b]Department of Medical Photonics, Graduate School of Medical Science, Kyoto Prefectural University of Medicine, 465 Kajii-cho Kawaramachi-Hirokoji, Kamigyo-ku, Kyoto, 602-8566, Japan

[*]E-mail: tminami@koto.kpu-m.ac.jp

Supplementary Table S1 Prediction results for tissue species with dimensional reduction of PCs. The right column PN (MN+UMN) indicates the sensitivities and specificities in which the peripheral nerves including myelinated nerve and unmyelinated nerve were predicted to be at least either myelinated nerve or unmyelinated nerve. MN, myelinated nerve; UMN, unmyelinated nerve; A, adipose tissue; C, collagenous tissue; SM, skeletal muscle; PN, peripheral nerve; PPV, positive predictive value; and NPV, negative predictive value.

Dimensional reduction of PC1

	Histology					
Prediction	MN	UMN	A	C	SM	PN (MN+UMN)
MN	957	19	1	2	4	1855
UMN	25	854	37	109	88	
A	0	0	962	0	0	0
C	15	59	0	881	14	74
SM	3	68	0	8	894	71
Sensitivity (%)	95.7	85.4	96.2	88.1	89.4	92.8
Specificity (%)	99.4	93.5	100	97.8	98.0	92.0
PPV (%)	97.4	76.7	100	90.9	91.9	88.5
NPV (%)	98.9	96.2	99.1	97.0	97.4	95.0

Dimensional reduction of PC2

Prediction	Histology					
	MN	UMN	A	C	SM	PN (MN+UMN)
MN	956	16	6	2	8	1818
UMN	26	820	148	78	95	
A	0	3	825	28	43	3
C	15	108	21	881	9	123
SM	3	53	0	11	845	56
Sensitivity (%)	95.6	82.0	82.5	88.1	84.5	90.9
Specificity (%)	99.2	91.3	98.2	96.2	98.3	88.8
PPV (%)	96.8	70.3	91.8	85.2	92.7	84.4
NPV (%)	98.9	95.3	95.7	97.0	96.2	93.6

Dimensional reduction of PC3

Prediction	Histology					
	MN	UMN	A	C	SM	PN (MN+UMN)
MN	884	17	1	0	105	1730
UMN	67	762	15	101	255	
A	0	0	978	0	0	0
C	6	35	0	882	173	41
SM	43	186	6	17	467	229
Sensitivity (%)	88.4	76.2	97.8	88.2	46.7	86.5
Specificity (%)	96.9	89.1	100	94.7	93.7	84.1
PPV (%)	87.8	63.5	100	80.5	65.0	78.4
NPV (%)	97.1	93.7	99.5	97.0	87.5	90.3

Dimensional reduction of PC4

Prediction	Histology					
	MN	UMN	A	C	SM	PN (MN+UMN)
MN	844	146	10	0	13	1875
UMN	156	729	20	98	98	
A	0	0	970	0	0	0
C	0	69	0	902	16	69
SM	0	56	0	0	873	56
Sensitivity (%)	84.4	72.9	97.0	90.2	87.3	93.8
Specificity (%)	95.8	90.7	100	97.9	98.6	92.0
PPV (%)	83.3	66.2	100	91.4	94.0	88.7
NPV (%)	96.1	93.0	99.3	97.6	96.9	95.7

Dimensional reduction of PC5

Prediction	Histology					
	MN	UMN	A	C	SM	PN (MN+UMN)
M	957	23	19	0	2	1644
UM	42	622	5	86	68	
AD	0	0	964	0	0	0
CT	0	298	0	823	45	298
SM	1	57	12	91	885	58
Sensitivity (%)	95.7	62.2	96.4	82.3	88.5	82.2
Specificity (%)	98.9	95.0	100	91.4	96.0	94.0
PPV (%)	95.6	75.6	100.0	70.6	84.6	90.1
NPV (%)	98.9	91.0	99.1	95.4	97.1	88.8
